# Evolving population distribution in China’s border regions: Spatial differences, driving forces and policy implications

**DOI:** 10.1371/journal.pone.0240592

**Published:** 2020-10-19

**Authors:** Daquan Huang, Yue Lang, Tao Liu

**Affiliations:** 1 School of Geography, Faculty of Geographical Science, Beijing Normal University, Beijing, China; 2 College of Urban and Environmental Sciences, Peking University, Beijing, China; 3 Center for Urban Future Research, Peking University, Beijing, China; Institute of Geographic Sciences and Natural Resources Research (IGSNRR), Chinese Academy of Sciences (CAS), CHINA

## Abstract

The security and socioeconomic development of China’s border areas are of great significance to the nation and the wider world. Using census, statistical, digital elevation model (DEM) and network data, this paper employs visual analysis to capture population distribution patterns in China’s 131 border counties from 1982 to 2010. Multiple stepwise regression is carried out to identify the influencing factors of population dynamics in border regions. The main findings include: China’s most heavily populated border areas are primarily in the northeast, northwest, and the Guangxi-Yunnan region, while rapid growth of population is found in western Inner Mongolia, southwest Xinjiang, northwest Tibet, and southern Yunnan. Given the increasingly market-oriented migration mechanism, the national reclamation policy has been no longer effective in population attraction in the new century. Education has significantly lowered and will continuously lower the fertility rate in remote border areas. The factors influencing population growth show a remarkable regional heterogeneity along China’s long border.

## 1. Introduction

Due to the increasing globalization and regional economic integration, sub-regional cooperation has emerged as there is now significant collaboration between neighboring countries [1,2]. The connotations and functions of national boundaries are shifting because this sub-regional cooperation involves border areas [3–6], which have become a relevant research topic. The traditional studies on border areas hold that they, as the boundary of two sovereign states, divide two economic systems, extend the space and time distance of interaction between economic actors, hinder the free flow of production factors, and bring difficulties to cross-border economic cooperation [7,8]. However, with the emergence of the economic belt in the border areas among European countries, the United States, Canada, and the United States and Mexico, scholars believe that the economic cooperation between the border areas will gradually get rid of the traditional regional mode of border trade and will be transformed into border development zones [9]. The *screening effect* of national borders is gradually weakening, whereas their *mediating effect* is becoming increasingly important [10,11]. The traditional division of people and economic activity by national boundaries has been replaced by a new world order in which the spatial flow of capital, industry, information, and population is no longer effectively impeded by territorial boundaries [12,13]. The border areas will become an important bridge for economic activities between countries [14].

However, this is accompanied by a series of border issues, such as illegal immigration, cultural conflicts, and so on [15–18]. Due to their special geographic locations, multi-ethnic settlements, and territorial disputes with nearby states, border areas have become politically and economically sensitive. In the border areas, all kinds of contradictions blend [19]. In a globalized world, border areas still play an important role in helping to distinguish different countries/regions and restrict the random flow of people. At present, China has four border trades, three sub-regional cooperations, and 14 border economic cooperation zones in the northeast, northwest, and southwest. The unprecedented opening of the country’s national frontiers has led to an increase in research on the topic. Recent studies on border areas have focused on the aspects of the socioeconomic growth (such as the border effect, economic cooperation zones, cross-border ethnic groups, and illegal immigration) [6,19–27].

People are the carriers of all economic activities and the basic force of social and economic development [28]. As the main factor of production, population migrates frequently between regions especially in the era of new-type urbanization [29,30], which makes the population spatial pattern in border areas quietly change and brings a series of effects on border security and economic and social development in border areas. Depopulation in China’s border areas threatens the national security [22]. At the same time, spatial differences in population distribution, structure, migration, and development are closely related to spatial differences in local conditions [31–34]. The distribution of the population and the influencing factors of the distribution change in border areas will be different than in other areas due to their unique location characteristics and border policies. Existing research has overlooked the issue of population distribution in these regions. Only a few studies have focused on smaller zones, shorter time scales, and borderlines [26,27,35–37]; or they considered population distribution and change within one kilometer of each side of the border [38]. As there is no administrative division within the study of 1 km of the frontier, it is difficult to propose specific policies for population distribution patterns and changes. The reasons for these shifts are mostly discussed from the perspective of sociology, which cannot provide a reference to create a national border policy. Hence, it is necessary to explore population distribution and influencing factors in China’s border regions in depth.

Given the shortcomings of existing research, this paper selects the county unit as the research object based on census data from 1982, 1990, 2000, and 2010, as well as each county’s statistical yearbook. The first aim is to examine population patterns in different counties and districts along the nation’s borders over a long period of time. The second goal is to analyze the factors affecting population changes in the state as a whole and regionally. Finally, the paper provides suggestions and references to develop population policies.

The novelty of this study lies in two aspects. First, the choice of the research scope: border areas. Data from eight 131 borderland counties, provinces, and regions are used for the analysis, and we added three dummy variables related to border policy, so that we can highlight the particularity of border areas. The differences between various borderland zones are effectively distinguished so that regional variations can reflect their unique characteristics. Furthermore, distinct orientations and directions can be identified in economic and social expansion to guide actively and effectively the characteristic development of each borderland area. Second, we carry out regression analysis of the factors affecting population change and the main elements are identified; such information can lead to more precise policy recommendations for healthy growth in border regions. The spatial heterogeneity in factors is also discussed by mapping the factors and comparing them with population geography.

The paper explores existing research on population distribution, population change, and border areas. Then, it introduces the general context of the research area, data selection and processing, and research methods. After presenting the study’s findings, the paper offers suggestions for the growth of border counties in different regions.

## 2. Literature review

### 2.1 The study on border areas

Border and boundary are two different concepts. A border divides national territories, whereas a boundary encompasses specific distances on both sides of the border [39]. Ratti (1993) considers a border as either a cross-border or marginal zone from the perspective of location [8]. After World War II (WWII), trade between European and North American countries became more frequent, especially following the establishment of the European Union (EU) and the North American Free Trade Agreement (NAFTA) in the early 1990s. An economic border belt gradually emerged between European and North American States, attracting academic attention. Some scholars believe that the existence of the border hinders the flow of production factors in the border areas and restricts the economic and social development of the border areas [7,8,40]. Others believe that border areas are an important bridge to carry out economic and trade activities between countries, mainly engaged in cross-border trade. Compared with the limited role of border areas in economy and trade, border areas are more conducive to the cooperation between a country, a region and a locality. The economic and cultural exchange between regions can promote the economic and cultural development of the border and surrounding areas [14,41]. Most foreign studies on border areas focus on the border effect, economic cooperation, illegal immigration, and other aspects [42,43]. Some explore the model of international cooperation between critical states [8,42]. Some scholars analyze the development of geo-economy from the perspective of economy [44]. Since the 1990s, China has gradually implemented an opening-up policy along its frontiers. Chinese research on border zones also encompasses studies on the border effect [10,11,23], border security (including theoretical discussions and identifying influencing factors) [45,46], economic cooperation and development, and sub-regional synergy [1,2,24].

Scholars have also studied the border population, which is the carrier of economic and social activities in border areas. West academics have primarily examined frontier populations in terms of cross-border movement and population security [16,18]. Stanley Hoffmann and Stephen Castles studied the national or regional security problems brought about by cross-border population mobility. Barry Zellen studied American border immigration and its border security. Katja Mirwaldt analyzed the interrelationship of cross-border ethnic groups from the perspectives of connection, conflict and geography [15–18]. Chinese scholars have conducted many studies on frontier residents. At present, the research on such communities includes five key aspects: (1) their distribution characteristics [38]; (2) population outflow and its influencing factors [26,27,35–37]; (3) population security’s impact on national security, as well as economic and social development [22,25]; (4) local inhabitants’ marital status [47,48]; and (5) illegal cross-border migrants [20,49]. However, most scholars focus only on some counties. You made use of 1 km grid data on population density in China’s border regions to analyze population changes from 2000 to 2015. However, due to the absence of administrative divisions, it is challenging to establish corresponding development measures for border counties and regions; furthermore, there is a lack of discussion on the factors that drive population shifts.

### 2.2 The spatial distribution of population

The mechanisms behind population distribution are critical. Population spatial patterns represent the population process in space. Moreover, population migration and flow are the primary aspects affecting population spatial patterns. Based on examinations of population migration abroad, hypotheses such as Lavingstein’s migration law, Berg’s push-pull theory, and the Lewis model have emerged. The study of population distribution and change in China is similar to other countries. Some scholars have investigated the natural growth of populations in different regions of China using data on population age structure and fertility rates [50–52]. As for the mechanical expansion of the population, many scholars have employed the push-pull theory to explain the causes of population migration flow [53,54]. Current research on the factors influencing population change includes:

1. Natural conditions: The influence of topography on population distribution is mainly caused by heat change of altitude, which affects people’s production and life, and finally affects population distribution. Ninety percent of the world’s population lives below 400 meters above sea level.
2. Economic factors: Some scholars have pointed out that the natural environment is the long-term factor affecting population spatial patterns, and economic components comprise the chief reasons for short-term changes in such patterns [55]. Some believe that economic growth has a decisive impact on population distribution and the dispersion or concentration of population distribution depends on economic development, especially shifts in the productivity level [39]. Industrial structure can also affect regional population growth through the different absorption capacities of various industries in the labor force [56].
3. Location conditions and policy factors: Previous studies have shown that location conditions and policy factors are the key features affecting population mobility [57]. Some scholars have proposed that geographic cost is a vital element that influences population distribution [58].
4. Public services: Regional differences in medical, health, and educational circumstances can directly affect population migration flow and growth [56,59]. Scholars have employed utility theory in applied economics to establish the behavioral analysis model of population flow and believe that the education level influences the behavior of the floating population.

In sum, existing studies on border areas mainly focus on economic functions, such as cross-border cooperation in border areas. Studies on the population in border areas mainly focus on the causes and effects of cross-border ethnic minorities, drug dealers, and other special groups, the movement of border population and other unique phenomena. However, there are few studies on the spatial distribution of population in border areas and most of them are limited to specific regions. At present, the situation of China’s border area population is complex, the structure is diverse, and it presents a different spatial agglomeration state. Border areas have the dual functions of opening to the outside world and guarding the border. The transformation of the population’s spatial structure is the key to optimize border economy, maintain border security, and realize national unity. Therefore, it is necessary to discuss the distribution, evolution, and influencing factors of population’s spatial patterns in border areas. In addition, to facilitate the implementation of policy recommendations, county administrative units are selected in this study as the research object.

## 3. Study area, data, and methods

### 3.1 Overview of the study area

**Fig 1:**
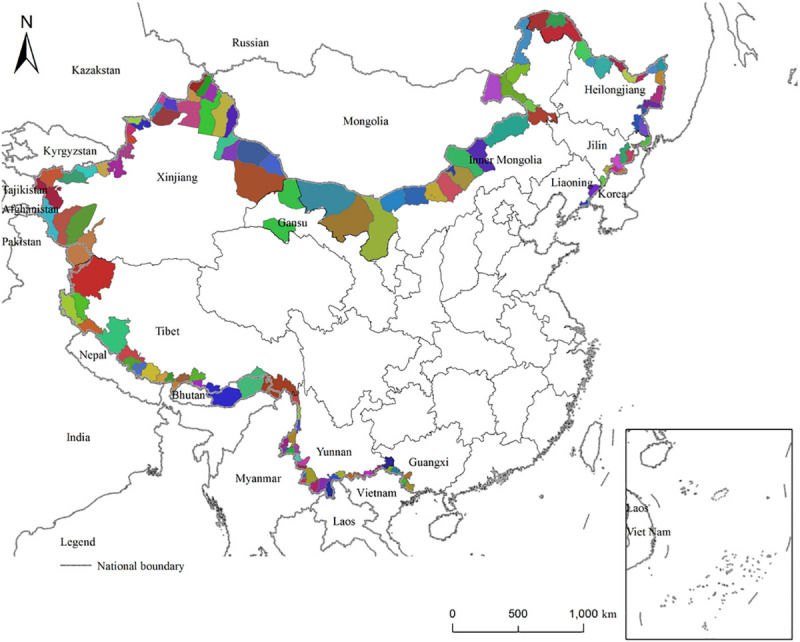


The study area comprises a national frontier that starts from the Yalu River estuary in the city of Dandong, with Liaoning Province in the east, and ends at the northern bay of the city of Fangchenggang, in Guangxi Zhuang Autonomous Region in the south (Fig 1). The total length is about 22,000 km, and 19,000 kilometers fall under ethnically autonomous areas. This frontier borders 14 countries, and more than 30 nationalities coexist. China’s borderland regions involve 9 provinces (autonomous regions), 41 cities (prefectures, regions, and leagues), and 136 border counties. Due to changes in the administrative divisions and the lack of data, the cities of Aihui and Suifenhe in Heilongjiang, Zhen'an and Yuanbao in Liaoning and Fangcheng in Guangxi were removed from the sample and 131 border counties were included in the research. China’s border areas are multi-ethnic, containing more than 30 of the country’s 55 ethnic minorities. In 2010, China’s border counties had a total population of 23.58 million people, among which nearly half were ethnic minorities. Being key zones of trade, cities, and ports, these locales have increasingly manifested their functions in terms of foreign commerce, circulation, geographic orientation, and openness to the outside world. Such features have led to high population mobility. In recent years, with the development of the Belt and Road Initiative, the China–Pakistan Economic Corridor, and the Association of Southeast Asian Nations (ASEAN) Free Trade Area (AFTA), China’s sub-regional synergy and border openings have reached an unprecedented peak. Population stability and economic prosperity in the country’s border zones can also provide experience for benign growth of population, economy and so on in other border areas.

China's inland boundary can be roughly divided into three sections, namely the northeast boundary line, the northwest boundary line and the southwest boundary line. The three border sections correspondingly form four border trade areas, namely the northeast border trade area, the Inner Mongolia border trade area, the northwest border trade area and the southwest border trade area [48]. At the same time, based on altitude and population density, the southwest border zone is composed of Tibet, Guangxi, and Yunnan. China’s border counties are roughly divided into the five regions listed in Table 1, according to which 23.58 million people live in the nation’s border counties, accounting for 1.69% of the overall population. The region is 2.1292 million square km, comprising 22.18% of China’s total land.

**Table 1:** Population, area, and distribution of China’s border counties in 2010.

Border region	Number of border counties	Population (1×10^4^)	Area (1×10^4^ km^2^)	Population density (people/ km^2^)	Proportion of the national population	Ratio to national population density
All	136	2358.00	212.92	11.07	0.0177	0.0800
Liaoning, Jilin, Heilongjiang	33	758.56	20.06	37.81	0.0057	0.2732
Inner Mongolia	19	188.84	62.00	3.05	0.0014	0.0220
Xinjiang and Gansu	33	498.27	68.46	7.28	0.0037	0.0526
Tibet	18	33.35	51.77	0.64	0.0003	0.0047
Guangxi and Yunnan	33	878.97	11.03	79.69	0.0066	0.5758

As seen in Table 1, the population density of China’s border counties is generally lower than the national average, at only 8%. At the same time, the population density of Guangxi and Yunnan, the highest among all border counties, is still just 58% of the national average.

### 3.2 Data selection and processing

To show statistical distribution patterns and population changes, this paper relies on data from the 1982, 1990, 2000, and 2010 population censuses in China’s border areas, (mainly containing information on the population, the illiteracy rate, and the proportion of ethnic minorities in the counties and districts along the frontier). Given that massive internal migrants have not changed their household registration, resident population can better reflect the real population of places than registered population in China. Census data are the only reliable source of information about the resident population at the county level. This is also the main reason for our selection of the four years when the latest four rounds of national censuses were conducted to do this research.

By reviewing relevant studies on population distribution, the factors affecting regional population distribution are identified. These include natural conditions, economic aspects, public services, science and education, and social elements. Therefore, data on traffic, the Average growth rate per annum of GDP, illiteracy, and employment in border counties and districts are collected to determine the elements that drive changes in population distribution. In addition, we also collected data related to national policies, such as the presence of border ports, border economic cooperation zones, and reclamation and border defense.

All information was obtained using county statistical yearbooks, 2010 road vector data, and digital elevation model (DEM) data either directly or through calculation.

### 3.3 Methodology

#### 3.3.1 Spatial distribution patterns and population changes in China’s border areas

Using 1982, 1990, 2000, and 2010 census data, this study illustrates population size, density, and shifts, as well as the absolute population change in border counties compared to local cities. The census results are visualized using ArcGIS software to analyze the spatial distribution patterns of China’s border communities. To identify the spatial distribution patterns in these locales, these census data are employed to contrast shifts in the absolute and relative populations in the 131 border counties. Comparisons with each border county’s population change are included, as well as that of the city in which the border county is located.

Absolute change in population:

$$A_{1}=P_{i+1}/P_{i}$$ (1)

*A1* represents population growth rate, *Pi+1* indicates the border county population at the end of the period, and *Pi* refers to the border county population at the beginning of the period. If *A1>1*, there was population growth. If *A1<1*, the population decreased. If *A1 = 1*, there was no change.

Relative change in population:

$$B_{1}=\frac{\frac{P_{i+1}-P_{i}}{P_{i}}}{\frac{D_{i+1}-D_{i}}{D_{i}}}$$ (2)

*B1* signifies the relative change in population, *Pi+1* represents the final border county population, and *Pi* signals the initial border county population. *Di+1* refers to the population of prefecture-level cities at the end of the period, and *Di* indicates the population of such cities at the beginning of the period. If *B1>1*, there was relative population growth. If *B1<1*, there was a relative population decrease. If *B1 = 1*, the relative population remained the same.

#### 3.3.2 Factors affecting spatial distribution and population change

To examine the main factors affecting the changing spatial patterns of population in China’s border counties, we firstly use multiple regression including all variables that are theoretically or practically expected to have effects. Multiple stepwise regression is then introduced to identify the key variables. This technique establishes the quantitative relationship between multiple variables in linear or nonlinear mathematical models and analyzes them with sample data. This paper examines the relationship between population change and natural conditions and economic factors; hence, this approach was chosen.

The process of variable selection by stepwise regression includes two basic stages: (1) Remove the tested insignificant variables from the regression model; and (2) Introduce new variables into the regression model.

Step 1: For the p regression, for regression variables X_1_, X_2_…, X_P_, respectively make them and the dependent variable Y to establish the regression model with one variable

$$Y=\beta_{0}+\beta_{I}X_{I}+\varepsilon ,I=1,\ldots ,p$$ (3)

Calculate variable Xi. The corresponding regression coefficients of F are the values of the test statistics $F_{1}^{\left(1\right)},\ldots ,F_{p}^{\left(1\right)}$. Maximize the $F_{i_{1}}^{\left(1\right)}$, namely, $F_{i_{1}}^{\left(1\right)}=max\;\{F_{1}^{\left(1\right)},\ldots ,F_{p}^{\left(1\right)}\}$, for a given significant level of alpha. Record the corresponding threshold for *F*^(1)^. If $F_{i_{1}}^{\left(1\right)}>F^{\left(1\right)}$, Then, $X_{i_{1}}$ is introduced into the regression model. And I1 is denoted as the indicator set of selected variables

Step 2: The binary regression model of dependent variable Y and independent variable subset {X_i1_, X_1_},…}, {X_i1_, X_i1-1_, 1} {X_i1_, X_i1_ +,…{X_i1_,X_p_} is established (that is, the regression element of this regression model is binary); The number is p-1. Calculate the regression coefficient variable, which is the statistical value of the F test. Remember $F_{k}^{\left(2\right)}(k\notin I_{1})$. Choose the largest one of them remember $F_{i_{2}}^{\left(2\right)}$, and the corresponding independent variable footer is marked as i_2_:

$$F_{i_{2}}^{\left(2\right)}=max\;\{F_{1}^{\left(2\right)},\ldots ,F_{i_{1}-1}^{\left(2\right)},F_{i_{1}+1}^{\left(2\right)}\ldots ,F_{p}^{\left(2\right)}\}$$ (4)

For a given level of significance of alpha, whereby the corresponding threshold for F (2) and $F_{i_{2}}^{\left(1\right)}\geq F^{\left(2\right)}$, the X_i2_ variables are introduced into the regression model. Otherwise, terminate the variable introduction process.

Step 3: Consider the regression of the dependent variable to the variable subset {X_i1_, X_i2_, X_ik_} and repeat Step 2.

According to this method, one of the independent variables that has never been introduced into the regression model is selected each time until no variable is introduced after the test.

Because the issue we studied involve many independent variables, it is difficult to determine that all the pre-selected independent variables have significant influence on the dependent variables, and there is no guarantee that all the independent variables are independent of each other. Therefore, in the establishment of multiple linear regression equation, variables should be screened according to the contribution of each variable to the dependent variable, and those independent variables with small contribution and close relationship with other independent variables should be eliminated, so as to obtain a concise and stable regression equation. Therefore, stepwise regression analysis method is selected in this paper.

By conducting multiple stepwise regression analysis of the elements that drive population change in border areas using SPSS software, the factors affecting the absolute population change in each county are identified.

The literature review points to the components that impact population shifts in border zones, which include the natural environment, economic development, geographic conditions, infrastructure construction, culture, education, and national macro policies.

As for the influence of natural conditions, we choose the index of average altitude, which represents the terrain conditions. The annual growth rate of GDP indicates the region’s economic performance. In terms of location, the distance from a prefecture-level city and a provincial capital city is selected to represent the degree of traffic access in the region. The average proportion of the minorities in 2000 and 2010 is selected to represent the ethnic structure in the county (Table 2). The change of illiteracy rate represents the improvement in the region’s cultural and educational level.

**Table 2:** Speed setting table for calculating cost distance.

Road type	Name	Type	Speed(km/h)
Road	highway	/	120
	main road	the national highway	100
		not the national highway	80
	general road	/	80
	dirt road	/	30
Railway	common rail	/	120

Border area is crucial for national security for its frontier location and ethnic diversity. To guarantee the border security and social stability, national policies are often introduced to stimulate economic development in the border area, leading to population growth. Three dummy variables are included in our model to investigate the effect of these policies, namely whether the border county has a border port, whether it is a border economic cooperation zone, and whether it has the national policy of reclamation and border defense.

Table 3 describes the data. The northeast border region and the Inner Mongolia border zone have a low average altitude, a small slope, a low degree of topographic relief, and relatively good topographic conditions. The average altitude and slope of Tibet’s border region are the highest among all border areas, and the terrain is poor. From 2000 to 2010, the Average growth rate per annum of GDP of each county and city in Inner Mongolia’s border zone was the highest, but regional development was not balanced and the differences were large. The Average growth rate per annum of GDP of the northeastern border region lags behind that of other areas. Traffic in Tibet and Inner Mongolia is poor, while the border counties and districts in the northeast, Guangxi, and Yunnan have better traffic access in their prefecture-level cities and provincial capitals. The ethnic minority population and illiteracy rate in Tibet’s border counties and districts are much higher than those in other regions, while in the northeastern border counties and districts, the opposite circumstances can be found.

**Table 3:** Description of selected independent variables in the regression analysis.

		All	Liaoning, Jilin, Heilongjiang	Inner Mongolia	Xinjiang and Gansu	Tibet	Guangxi and Yunnan
Population density 2000	mean	46.60	88.83	16.76	11.43	1.70	87.57
	Std dev	7717.37	24859.39	2884.68	168.08	1.87	1625.76
	max	682.14	682.14	237.69	61.50	4.08	181.20
	min	0.09	1.87	0.26	0.52	0.09	7.91
Population density 2010	mean	51.19	99.64	22.00	12.38	1.86	92.40
	Std dev	11500.50	38814.37	5501.55	192.55	2.08	2287.64
	max	823.33	823.33	327.40	65.13	4.34	242.50
	min	0.13	1.61	0.34	0.60	0.13	8.62
Population change, 2000–2010	mean	1.09	1.02	1.18	1.10	1.14	1.05
	Std dev	0.0402	0.0236	0.1777	0.0113	0.0095	0.0153
	max	2.33	1.64	2.33	1.42	1.36	1.34
	min	0.44	0.85	0.44	0.84	0.99	0.65
Altitude (m)	mean	1655.22	418.37	1032.56	1931.99	4579.02	1250.16
	Std dev	2145395	84962	77556	1195174	763584	427462
	max	5147.01	1074.58	1391.47	4662.37	5147.01	3076.75
	min	37.38	37.38	634.67	737.81	2204.33	84.77
Annual GDP growth rate, 2000–2010	mean	0.18	0.15	0.25	0.18		0.16
	Std dev	0.0047	0.0035	0.0056	0.0049		0.0005
	max	0.43	0.39	0.4	0.43		0.19
	min	0.05	0.09	0.13	0.05		0.10
Time to prefecture-level city (mins)	mean	21.32	12	24.1	18.87	30.4	11.38
	Std dev	122	43	63	104	94	14
	max	50.09	27.7	41.72	36.55	50.09	19.63
	min	1.33	1.33	11.46	4.94	11.31	39.66
Time to provincial capital (mins)	mean	53.67	37.55	49.51	75.29	64.65	33.45
	Std dev	653	316	386	1187	1049	154
	max	137.06	93.42	91.2	133.21	137.06	60.8
	min	12.37	14.69	22.28	25.73	18.59	12.37
Proportion of ethnic minorities	mean	53.47	13.17	35.37	65.16	94.31	67.76
	Std dev	1175	323	527	563	32	680
	max	99.78	58.13	75.36	99.78	99.15	98.73
	min	1.32	1.32	8.73	26.4	74.94	5.97
Change of illiteracy rate, 2000–2010	mean	0.49	0.46	0.38	0.36	0.63	0.66
	Std dev	1.15	0.51	0.41	0.95	0.60	1.08
	max	1.20	0.81	0.52	1.00	0.88	1.20
	min	0.0480	0.0137	0.0128	0.0311	0.0351	0.0626
Border port	count	63	18	8	15	8	14
	proportion	0.48	0.62	0.42	0.45	0.44	0.44
Border economic cooperation zone	count	19	7	2	3	0	7
	proportion	0.15	0.24	0.11	0.09	0.00	0.22
Reclamation and border defense	count	32	0	0	32	0	0
	proportion	0.24	0.00	0.00	0.97	0.00	0.00

## 4. Results

### 4.1 Population distribution and changes in borderland areas

#### 4.1.1 Large populations are located at Northeast and Southern provinces

**Fig 2:**
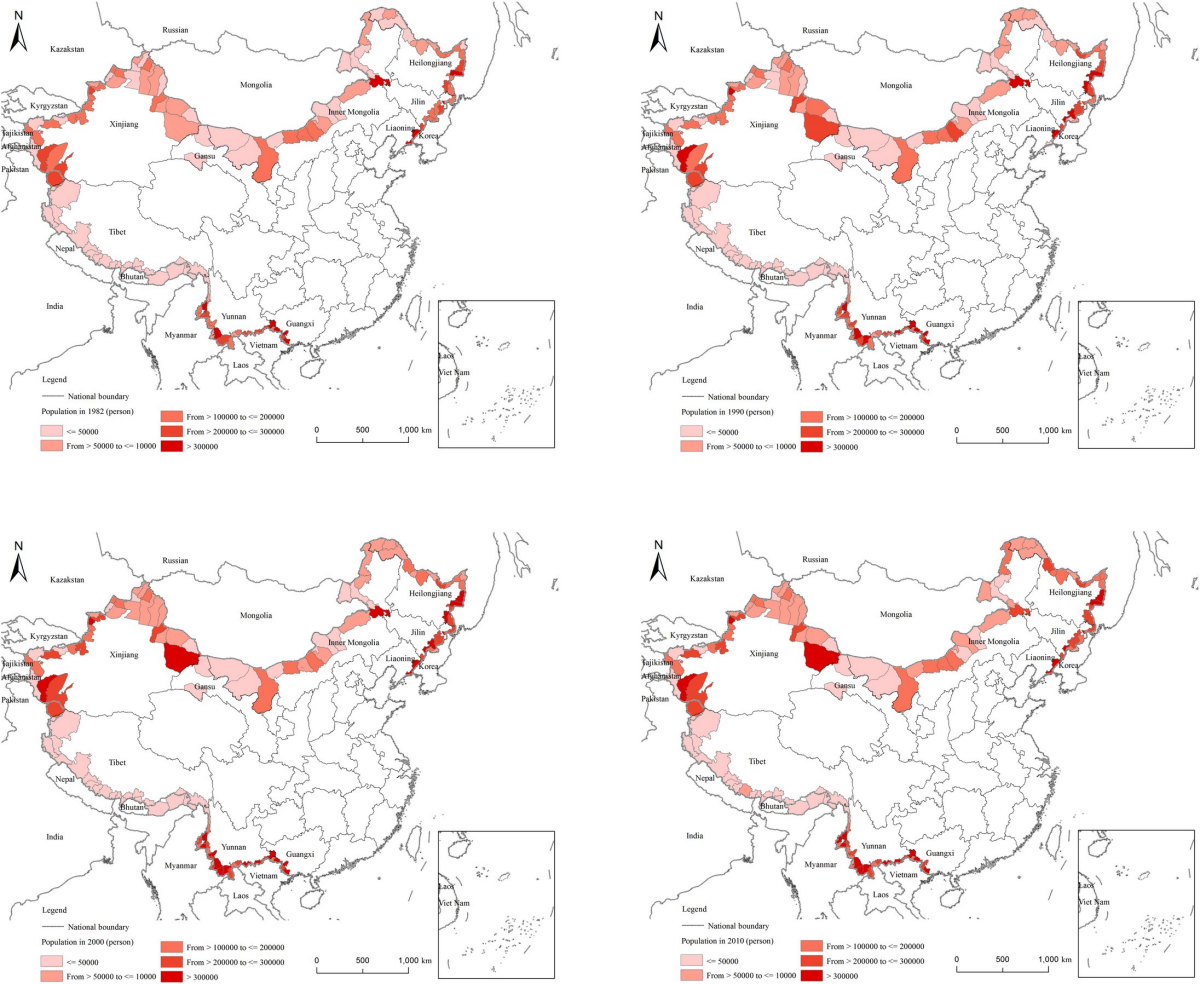


**Fig 3:**
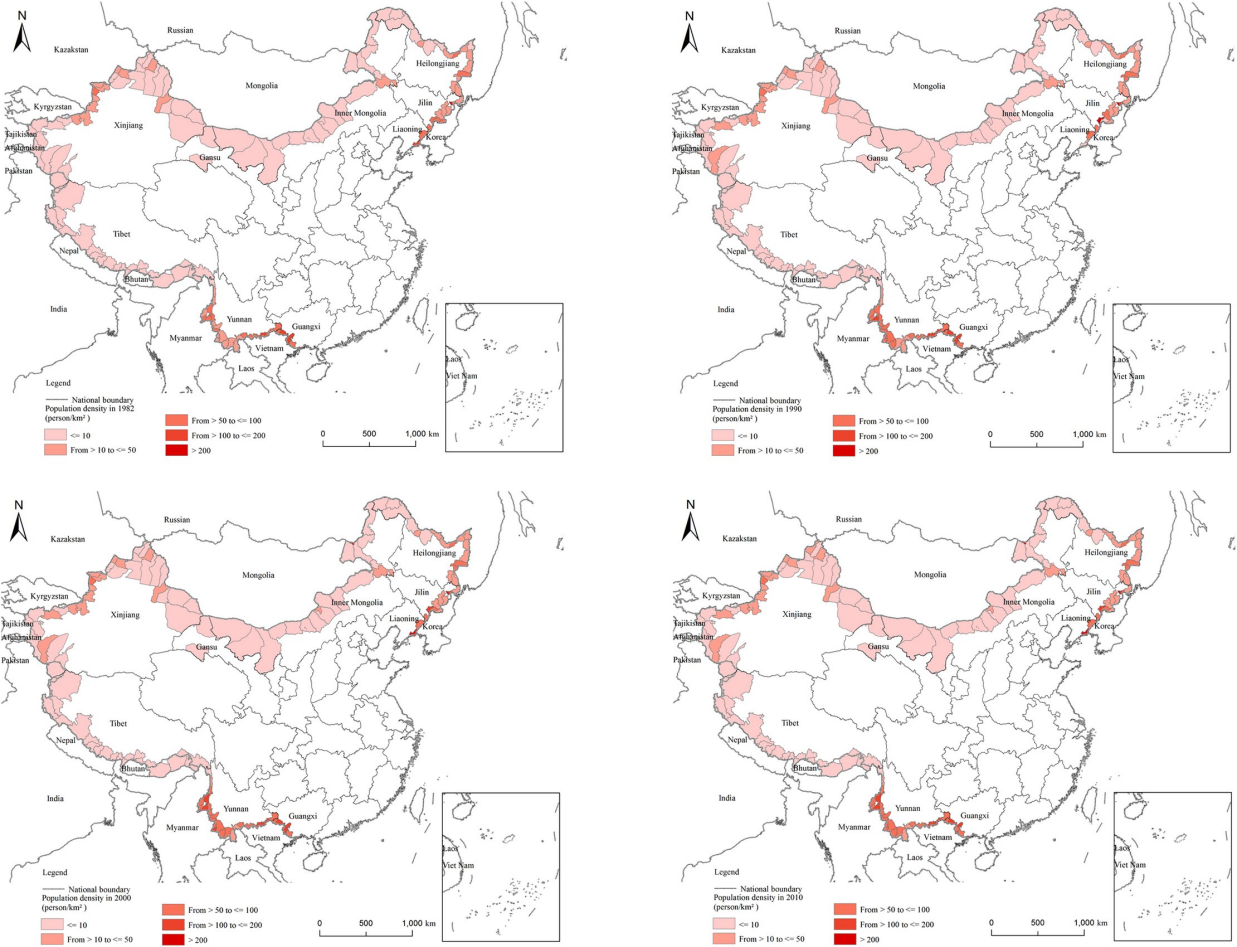


According to the national census, Fig 2 demonstrates that the population distribution of China’s border counties did not change much between 1982 and 2010. The population distribution basically conforms to the Huhuanyong Line. The large-scale counties are located in the three northeastern provinces and Xinjiang, Guangxi, and Yunnan (autonomous regions). The population density is similar to that of population quantity (Fig 3). The population density of Northeast China and Yunnan is clearly higher than that of other regions. Tibet, on the other hand, has a smaller population.

#### 4.1.2 Negative population growth is evident in northeast border counties, while the populations of western border counties are continuously increasing

**Fig 4:**
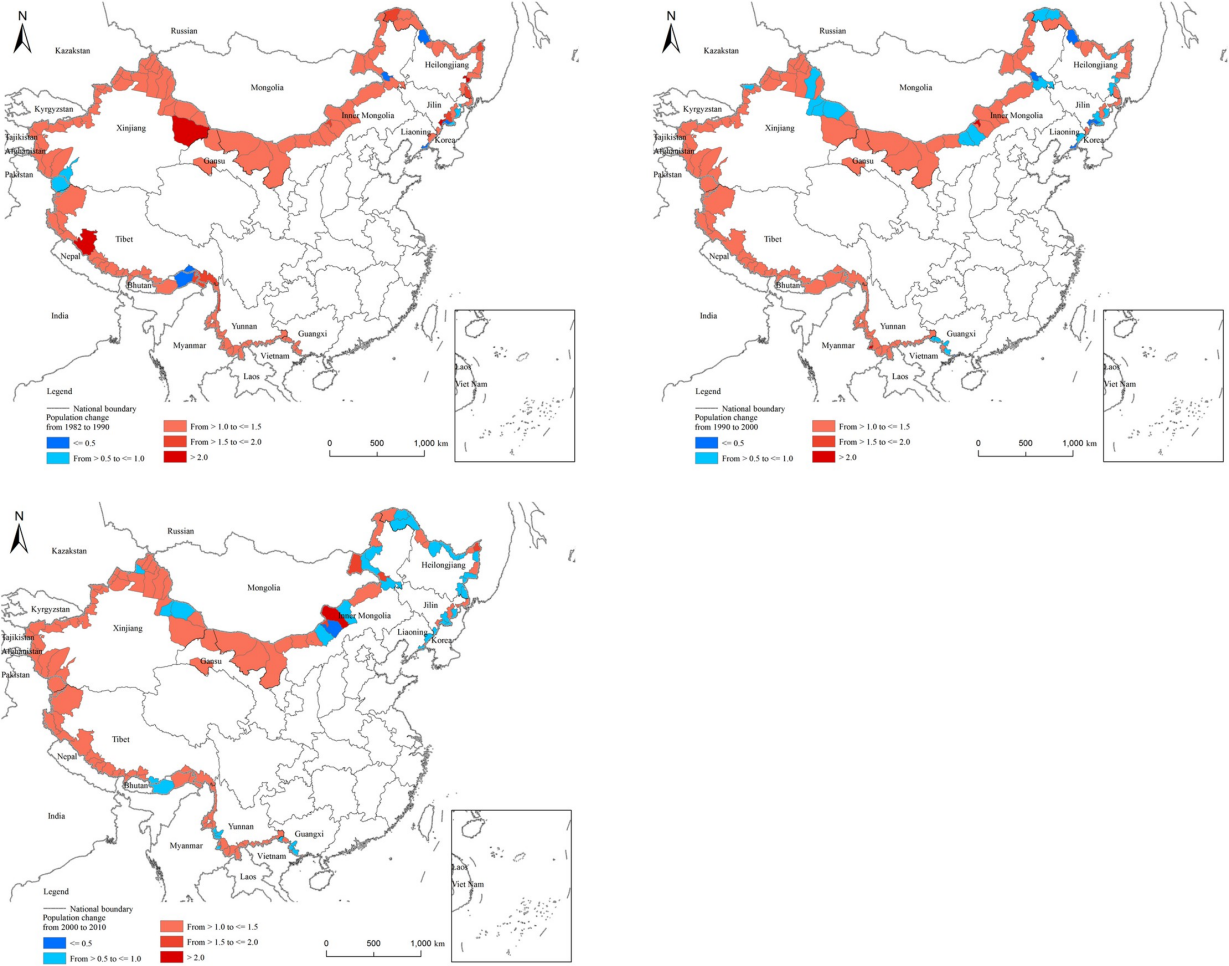


The Numbers obtained by dividing the population at the end of the period and the population at the beginning of the period in each county reflect the change of the population during this period. There is a large difference between the population change and the distribution pattern (Fig 4). From 1982 to 1990, the population increased in most regions, and the growth rate was not very different; only a few regions saw a decline in population. From 1990 to 2000, the populations of border counties in the west, as well as several border counties in Inner Mongolia, expanded rapidly. From 2000 to 2010, the border populations of Inner Mongolia, Xinjiang, and Tibet continued to rise, while the populations of the northeastern border counties and districts showed negative growth.

Table 4 indicates that the population growth rate in the border areas for each period was higher than the national average. Table 4 also shows that the population growth rates of Inner Mongolia and Tibet were higher, while that of the border counties in the three northeastern provinces was lower than the national average for all three periods.

**Table 4:** Changes in China’s border county populations.

	1982–1990	1990–2000	2000–2010
National average	1.1239	1.1085	1.0576
Border counties	1.2385	1.1604	1.0874
Liaoning, Jilin, Heilongjiang	1.3883	1.1063	1.0523
Inner Mongolia	1.1575	1.2974	1.1300
Xinjiang and Gansu	1.1946	1.1303	1.0997
Tibet	1.2409	1.1814	1.1358
Guangxi and Yunnan	1.2028	1.1483	1.0593

The value is the ratio of the population at the end of the period compared to that at the beginning.

**Fig 5:**
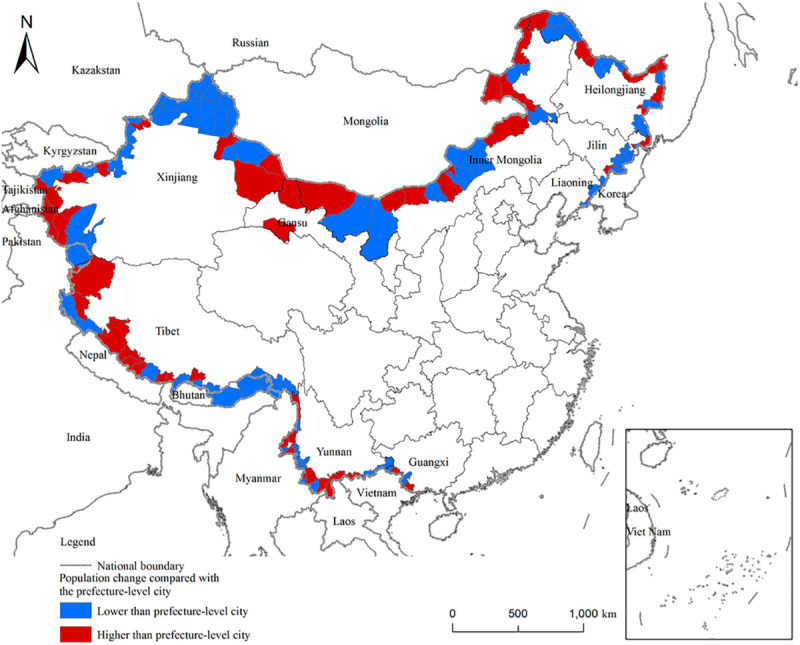


Each border county’s demographic shifts are compared to those of their corresponding cities for the 2000–2010 period. Next, the counties’ relative population changes are estimated. In Fig 5, the red area signals that the region’s population growth was higher than its city location, while the blue area denotes the opposite. Fig 5 illustrates that population growth in the northeastern border areas, in comparison to their corresponding cities, generally lagged behind. This finding might be related to natural conditions, transportation, various policies, or other factors. This finding is analyzed in the following paragraphs.

### 4.2 Factors that influence population distribution patterns in border areas

Results of the regression model including all variables are shown in Table 5. Natural condition, ethnic structure, do not have significant influence on population density and change in total population of border area. The positive effect of economic growth emerged in 2010. The locational condition within a prefecture is more important than that within the province for population and its change in border counties. Better education is negatively associated with population growth indicating the lower fertility rate of educated population than illiteracies.

**Table 5:** Regression results of full variable models.

	Population density 2000		Population density 2010		Population change, 2000–2010	
	Beta	Sig	Beta	Sig	Beta	Sig
Altitude (m)	-0.01	0.45	-0.01	0.59	0.00	0.13
Annual GDP growth rate, 2000–2010	167.35	0.12	228.75	0.09	-0.10	0.74
Time to prefecture-level city (mins)	-3.66	0.00	-4.32	0.00	0.01	0.01
Time to provincial capital (mins)	-0.02	0.95	0.02	0.96	0.00	0.60
Proportion of ethnic minorities	0.05	0.85	0.01	0.98	0.00	0.72
Change of illiteracy rate, 2000–2010	-27.62	0.42	-47.82	0.26	-0.22	0.03
Border port	-2.63	0.86	-7.14	0.69	0.06	0.17
Border economic cooperation zone	45.52	0.03	58.33	0.02	0.02	0.76
Reclamation and border defense	-47.30	0.00	-56.06	0.01	-0.08	0.09
Constant	115.51		133.90		1.08	
R^2^	0.35		0.33		0.14	
F	7.37		6.47		2.27	

Effects of national preferential policies are notable and complex. The policy of reclamation and border defense is significant and negative associated with population density. It is reasonable because it is the less populated places that have the demand for such a migration policy to safeguard the territory security. However, the effectiveness of this policy has totally disappeared in the new century as indicated by the results. Migration in contemporary China is determined mainly by economic factors rather than political forces. Those who migrated to border regions for political reasons are re-migrating back to their hometown or other cities, which has led to a more severe condition of emigration in the border counties that historically experienced the reclamation and border defense policy. The border ports have not attracted immigrants in border areas over the study period. The positive relationship between population density and the establishment of border economic cooperation zones largely represent the locational preferences of the latter rather than its effectiveness in population attraction. This conclusion is proven by its insignificant association with population growth.

Multiple stepwise regression is then conducted to identify the key influencing factors of population flow in border counties. We can see from the result that the time to the prefecture-level city, illiteracy rate change from 2000 to 2010 and whether the county has the national policy of reclamation and border defense have significant effect on population change in border counties (Table 6). Travel time to the prefecture-level cities has a positive effect on population change which means that the more convenient the transportation between the county and the prefecture-level cities, the more likely the population of the county will decrease. It may be due to the fact that the more convenient the transportation between the county and the prefecture-level city, the more easily the people can move to the prefecture-level city or other more developed cities. The change of illiteracy rate is negatively correlated with population change. That means the more the illiteracy rate in the county decreases, the more the population of the county increases. In contrast to that of population change, factors highly associated with the population density which represents the stock population standardized by area do not include education but include economic growth and national policies. These differences are in accordance with the results of the full variable regression shown in Table 5.

**Table 6:** Results of stepwise regression models.

	Population density 2000		Population density 2010		Population change, 2000–2010	
	Beta	Sig	Beta	Sig	Beta	Sig
Altitude (m)						
Annual GDP growth rate, 2000–2010	208.007	0.038	284.018	0.024		
Time to prefecture-level city (mins)	-4.073	0.000	-4.676	0.000	0.05	0.001
Time to provincial capital (mins)						
Proportion of ethnic minorities						
Change of illiteracy rate, 2000–2010					-2.57	0.001
Border port						
Border economic cooperation zone	46.001	0.016	56.099	0.018		
Reclamation and border defense	-40.019	0.003	-44.162	0.009		
Constant	90.591		92.452		1.118	
R^2^	0.346		0.313		0.161	
F	16.656		14.367		12.303	

**Fig 6:**
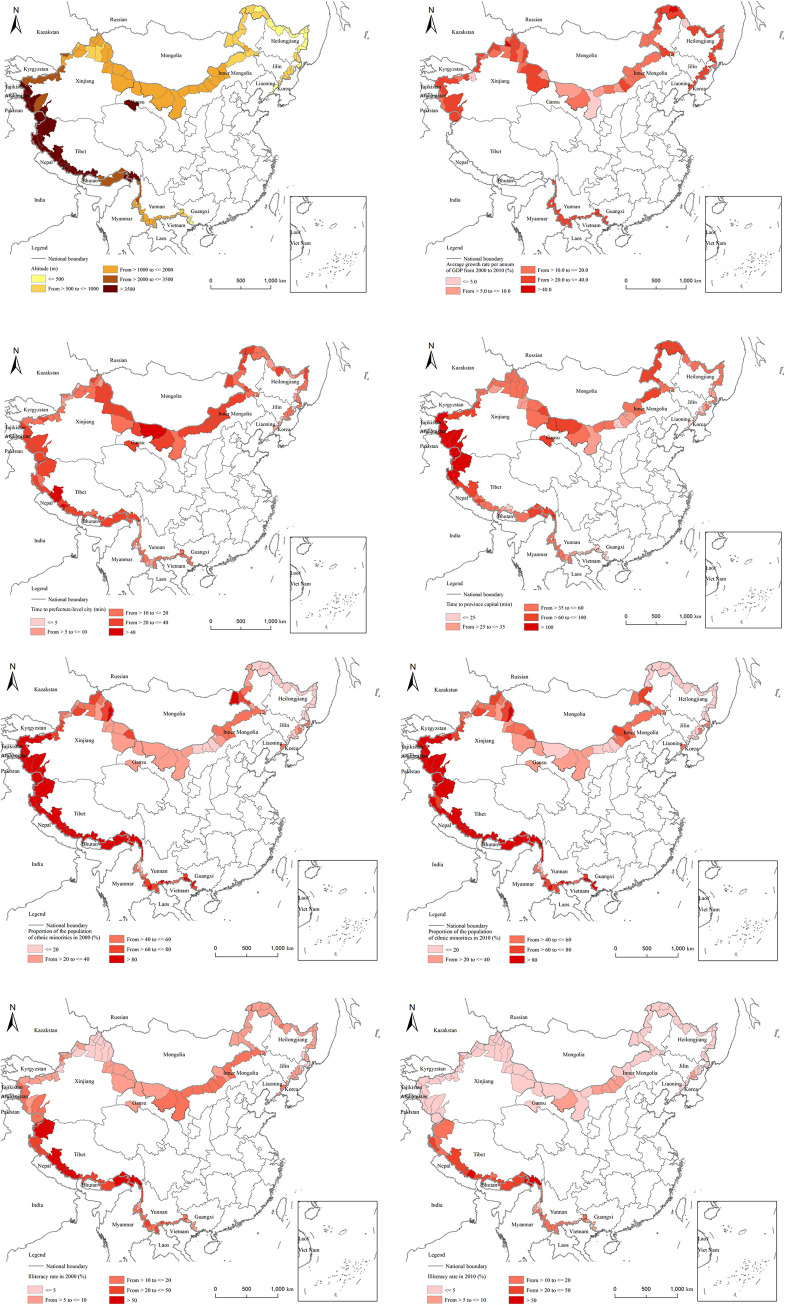


Result of the regression models indicates limited explanatory power of many independent variables. Nevertheless, it does not mean that these factors are not important in shaping the geography of China’s border population. When we compare the maps of changing population distribution with those of influencing factors shown in Fig 6, more local relationships between them can be identified. The recent trend of population changes after 2010 when the latest census data were available can also be revealed and discussed by this comparison.

#### 4.2.1 Northeast: Heilongjiang, Jilin, and Liaoning

During the research period, the population growth rate in northeast China was lower than the national average and kept slowing down. It is the case especially after entering the new century and will be continuing for a long time if not forever. The spatial distribution of the population change is uneven and the counties with a population decrease are obviously more than those with a population increase.

China’s northeast border refers to the northeastern border areas of Liaoning, Jilin, Heilongjiang, and the eastern Inner Mongolia Autonomous Region. The region is located in the northeast of China, adjacent to the DPRK, the Russian Far East and Mongolia. The northeast border area has many neighboring countries, making it convenient for developing economic ties with surrounding countries and overseas regions. In addition, the trade system and trade ports in the northeast border area are optimal, with dense cities and better traffic conditions. The topographic conditions, traffic, and the proportion of the minority population in each county in this region are similar; therefore, these factors have a small effect on population shifts. The changes in GDP and the illiteracy rate (that is, improvements in science, education, and culture) have a great impact on population shifts in this region. The main reason for the population decrease in northeast China is migration; compared with other border areas, the traffic conditions in northeast China are more developed, and people can move out more easily. To stabilize the population, corresponding measures should be taken to slow down the population emigration and attract the migrants.

#### 4.2.2 North: Inner Mongolia

In the border areas of Inner Mongolia, the population of most counties increased, the economic growth rate was relatively high in most counties, and the proportion of the ethnic minorities was not much different across counties. Therefore, these factors have no significant influence on the spatial variation of population change. However, the difference in altitude is large, which has a deep effect on regional population changes. Variations in traffic and education also have a considerable influence on the regional geography of population change.

Looking at the counties with declining populations, most of them have poor natural conditions and traffic conditions, such as Prairie Chenbarhu banner, which has Hulunbuir Sandy Land in its county area and only one national highway. The loss of population was exacerbated by the poor environmental and infrastructure conditions. In contrast, there was a marked increase in population in Manzhouli and Erenhot. These two counties are the main port cities of Inner Mongolia, with perfect transportation systems and foreign trade systems.

#### 4.2.3 Northwest: Gansu and Xinjiang

The population increased in most border counties in the northwest. The proportion of the ethnic minorities in the region does not vary much; also, the illiteracy rate has significantly reduced but is relatively balanced in the region. Therefore, these factors have no substantial effect on population shifts. The altitude of each county varies greatly and the level of economic development is clearly different. Hence, topographic conditions and economic growth have an influence on population changes in border counties. Traffic varies greatly. Also, due to the regressive development of prefecture-level cities in the region, traffic access between the region and provincial capitals has a deep impact on population change. These counties have the national policy of reclamation and border defense, which is expected to help them in regional development and population attraction. However, the regression results show that such an relationship may not exist and the changing population geography may have little to do with this policy.

#### 4.2.4 Southwest: Tibet

We can see that the populations of almost all border counties in Tibet increased in the study period. China has proposed many special preferential policies in Tibet, including migration of cadres, investment in infrastructures and social welfares, and all types of economic stimulation policies. These national polices have direct and indirect effects on population changes in Tibet. For example, the degree of traffic access has important influence on regional population change. The opening of the Qinghai-Tibet Railway and Nyingchi airport, Ali Airport, and Xigaze Airport have laid a strong foundation for the development of border counties in Tibet, which is convenient for them to develop border tourism. In recent years, tourism has become an important industry in Nyingchi, Shannan, Xigaze, and Ali. Moreover, the unique cultural, religious and linguistic barriers between Tibetan and Han people have also hindered emigration of Tibetan. All these factors contribute to population concentration and growth in Tibetan border counties.

#### 4.2.5 South: Guangxi and Yunnan

In the past decade, the economic development of this region has lagged slightly behind the overall level of the border; but the difference is not significant. In other words, economic growth in this region is relatively balanced; thus, the impact of economic aspects on the region’s population is not apparent. Due to the region’s dense population, the area of border counties here is considerably smaller than that in other regions and they often have a higher degree of traffic access to the prefecture-level cities in which they are located. Hence, the traffic access to the prefecture-level city greatly influences population changes in this region. In addition, border trade and tourism are industries with special advantages in border counties in this region, which is an important factor to attract labor force. The populations of Ruili, Dongxing, and other border port cities and tourist cities have increased significantly in recent decades.

## 5. Conclusion, discussion, and policy recommendations

In terms of population distribution and change in border areas, from 1982 to 2010, the population distribution of China’s border counties did not vary much. Counties with large populations are generally located in three provinces in northeast China, i.e., Xinjiang, Guangxi, and Yunnan (autonomous regions), while Tibet has a smaller population. There are great variations between population shifts and distribution patterns. The border populations of Inner Mongolia, Xinjiang, and Tibet continued to rise. The growth rate of populations in border areas during each period is higher than the national average, while the growth rate of the populations in Inner Mongolia and Tibet is higher. The population growth rate of border counties in the three northeastern provinces from 1990 to 2010 is lower than the national average.

Given the increasingly market-oriented migration mechanism, the national reclamation policy has been no longer effective in population attraction in the new century. Education has significantly lowered and will continuously lower the fertility rate in remote border areas. In addition, factors influencing population growth show a remarkable regional heterogeneity along China’s long border. For example, emigration are important in the population change of border counties in the northeast, just like traffic and education in the north, topographic conditions and traffic in the northwest, national political and financial support in the southwest and border trade and tourism in the south.

According to the different population distribution patterns and changes in the border areas, as well as the analysis of the factors affecting population distribution in these locales, the following suggestions are put forward.

Migration as the main reason of population change has been dominantly determined by market forces rather than political ones in current China. In this sense, investment and economic development should be viewed as the key measure to prevent population decline and promote population growth in border counties. The level of economic development in border areas lags behind that of the national average. It may indicate a possibly higher return of investment. But the state should continuously improve the traffic and infrastructures in these remote areas and propose more open economic and trade policies to guarantee such a high return. Moreover, direct financial support on social welfares of residents in border areas is also important because these social resources are key for preventing population outflow and attracting people from the outside.

To be specific, the following financial and policy support are highly recommended. Taking advantage of opportunities such as the Belt and Road Initiative, Western development strategies and other regional strategies that are closely related to border areas should be integrated more deeply into the national opening up strategy. Port cities should be developed, and large urban centers should be built with nodes for international exchanges. Border zones’ special geographic advantages should be exploited to foster trade between these areas and neighboring states. It is necessary to continuously improve infrastructure in border areas and raise the level of public services in border areas, such as transportation, medical and health care, and science, education and culture. In this way, the living standard of residents in border areas can be improved, and meanwhile, human and material resources in border areas can be ensured to flow rapidly and resources can be fully mobilized.

Current research still has some weaknesses. Due to limited data availability, the selection of independent variables is one-sided. For example, change in the illiteracy rate is only used to represent improvement in the regional level of culture, science, and education. Furthermore, the impact of regional development policies and public service level on population change is insufficient. At the same time, due to a lack of data, there is no information on shifts in Tibet’s annual GDP rate from 2000 to 2010, so it is impossible to analyze the effect of economic development on population changes in this region’s border counties.
